# A Verifiable Framework for Brain Tumor Classification: Combining Vision Transformers, Class-Weighted Learning, and SMT-Based Formal Decision Traces

**DOI:** 10.3390/diagnostics16091361

**Published:** 2026-04-30

**Authors:** Mehmet Akif Çifçi, Kadir Karataş, Fazli Yıldırım, Ali Doğan

**Affiliations:** 1 Institute of Research and Development, Duy Tan University, Da Nang 551111, Vietnam; 2 Department of Computer Engineering, Bandırma Onyedi Eylül University, 10200 Balıkesir, Türkiye; 3 Faculty of Economics, Administrative and Social Sciences, Department of Management Information Systems, Istanbul Topkapı University, 34087 Istanbul, Türkiye; 4 Department of Neurology, Faculty of Medicine, Bandırma Onyedi Eylül University, 10200 Balıkesir, Türkiye

**Keywords:** brain tumor, MRI, slice-level classification, deep learning, convolutional neural networks, Swin Transformer, external validation, symbolic auditing, SMT (Z3)

## Abstract

**Background/Objectives**: Automated brain tumor classification from MRI is particularly challenging when restricted to single post-contrast axial T1-weighted slices without volumetric or clinical context. **Methods**: We present a four-class (glioma, meningioma, pituitary tumor, no tumor) slice-level classification framework that combines a fine-tuned Swin-Tiny Transformer with inverse-frequency class-weighted learning and a prototype SMT-based symbolic auditing layer for post hoc logical consistency checks. All architectures were trained and evaluated under identical preprocessing, augmentation, optimization, and evaluation protocols. **Results**: On an internal clinical dataset from Bandırma Onyedi Eylül University Hospital (*n* = 8040 slices), Swin-Tiny achieved 97.42% slice-level accuracy (macro-F1 97.42%, macro-AUC 0.994), exceeding matched convolutional baselines by approximately eight percentage points. Five-fold stratified cross-validation confirmed stability (mean accuracy 97.40% ± 0.28%). Zero-shot evaluation on the independent BRISC-2025 dataset (*n* = 6000 slices) yielded 94.82% accuracy and macro-AUC 0.97, indicating maintained performance under acquisition-related distribution shift. Per-class metrics were consistently high across tumor types, with residual errors dominated by glioma–meningioma confusion, reflecting known radiologic overlap on single contrast-enhanced T1 slices. The symbolic auditing layer flagged 1.2–2.9% of predictions as constraint-violating; most such cases were borderline but correctly classified, suggesting sensitivity of heuristic thresholds rather than systematic model failure. **Conclusions**: These findings support the value of hierarchical shifted-window attention for integrating local texture and broader spatial context in slice-level MRI classification. While patient-wise, multimodal, and prospective validation remain necessary for clinical deployment, this study provides a controlled empirical benchmark and a prototype mechanism for post hoc logical auditing in neuro-oncologic imaging.

## 1. Introduction

Primary brain tumors constitute a heterogeneous group of neoplasms arising from glial, meningeal, pituitary, or related intracranial tissues, and they remain among the most clinically challenging malignancies due to their high morbidity, limited therapeutic options, and often poor prognosis [1,2]. Gliomas in particular account for a substantial fraction of malignant primary brain tumors and are associated with unfavorable survival even under aggressive multimodal treatment, whereas meningiomas and pituitary tumors exhibit more variable clinical courses that range from indolent to highly aggressive [3]. Accurate differentiation among these entities is therefore central to treatment planning, prognostic assessment, and longitudinal monitoring. In routine clinical practice, this differentiation relies heavily on magnetic resonance imaging, which provides detailed soft tissue contrast and visualization of lesion morphology, enhancement patterns, and surrounding anatomy [4,5].

Despite its central role, brain MRI interpretation is a cognitively demanding task that requires extensive neuroradiologic expertise. Diagnostic decisions are often made under conditions of uncertainty, especially when tumors share overlapping radiologic characteristics such as heterogeneous enhancement, mass effect, or proximity to dural structures [6]. Interobserver variability remains a persistent concern, particularly for borderline cases and for institutions with limited subspecialty coverage [7]. These limitations have motivated sustained interest in computer-aided diagnostic systems that can provide consistent, quantitative assessments of tumor appearance and reduce dependence on subjective visual judgment [8].

Recent advances in artificial intelligence, and deep learning in particular, have reshaped medical image analysis by enabling end-to-end learning directly from pixel-level data [9,10]. Convolutional neural networks have become the dominant paradigm for image-based tasks due to their ability to learn hierarchical feature representations through stacked convolutional and pooling operations [11]. In neuroimaging, CNNs have been applied to tumor detection, segmentation, grading, and multi-class classification, often achieving performance comparable to or exceeding traditional machine-learning approaches that rely on handcrafted features [12,13]. However, CNNs are inherently biased toward local feature extraction, and their ability to integrate spatially distant but semantically related regions depends on network depth and pooling structure. As a result, long-range contextual relationships within large field-of-view MRI slices may be only weakly modeled, particularly in relatively shallow or lightweight architectures [14,15].

Transformer-based vision models were introduced to address these limitations by replacing or augmenting convolutional operations with self-attention mechanisms that explicitly model interactions between spatial locations [16]. Vision Transformers and their variants have demonstrated strong performance across a wide range of computer vision tasks, including medical image classification and segmentation [17,18]. By computing attention weights over all image patches, these models can capture global dependencies in a single layer. However, naïve global self-attention scales quadratically with input size, which poses computational challenges for high-resolution medical images and may dilute fine-grained local details that are diagnostically important [19].

The Swin Transformer architecture was proposed as a hierarchical alternative that balances local detail preservation with global context aggregation through shifted-window self-attention [20]. By restricting attention to non-overlapping local windows and periodically shifting these windows across layers, Swin models achieve linear computational complexity with respect to image size while progressively expanding the effective receptive field. This design introduces an inductive bias that is well aligned with medical imaging, where both local texture cues, such as enhancement heterogeneity, and broader anatomical context, such as lesion location relative to known landmarks, contribute to diagnosis [21]. Prior studies have reported that Swin-based models outperform conventional CNNs and standard Vision Transformers on a variety of neuroimaging tasks, including brain tumor classification and segmentation, often with improved robustness to scanner and protocol variability [22].

Most existing work in brain tumor classification focuses either on volumetric three-dimensional MRI or on multi-sequence inputs that combine T1, T2, FLAIR, and diffusion-derived images [23]. While these approaches can exploit rich spatial and multiparametric information, they require complete patient studies, consistent acquisition protocols, and substantial computational resources. In contrast, many publicly available datasets and real-world deployment scenarios involve two-dimensional slices without reliable patient identifiers, volumetric continuity, or clinical metadata. Slice-level classification therefore represents a constrained but practically relevant problem setting, especially for large-scale retrospective analysis, weakly supervised learning, and benchmarking of architectural inductive biases [24].

Within this constrained setting, methodological rigor remains uneven across the literature. A significant number of published studies rely on a single train test split, lack stratified cross-validation, or omit evaluation on independent external datasets, which limits confidence in reported performance and generalization claims [25]. Furthermore, comparisons between CNNs and transformer-based models are often confounded by differences in preprocessing, data augmentation, or training protocols, making it difficult to attribute performance gains to architectural properties alone.

In this work, we conduct a controlled and systematic comparison of convolutional and transformer-based architectures for four-class brain tumor classification from contrast-enhanced axial T1-weighted MRI slices. In this work, we conduct a controlled and systematic comparison of convolutional and transformer-based architectures for four-class brain tumor classification from contrast-enhanced axial T1-weighted MRI slices. The study is grounded in two independent datasets that share an identical label space. The internal dataset comprises 8040 post-contrast axial slices acquired at Bandırma Onyedi Eylül University Hospital. The external dataset, BRISC-2025, contains 6000 slices collected under different acquisition conditions and serves as an independent generalization benchmark. Each sample corresponds to a single two-dimensional slice, and no patient-level grouping or volumetric context is available. All analyses therefore operate strictly at the slice level, a constraint that is explicitly acknowledged in both the experimental design and the interpretation of results.

We evaluate three model families under identical preprocessing and training conditions: a custom convolutional neural network trained from scratch, a deeper residual CNN with skip connections to improve feature learning stability, and a Swin-Tiny Transformer pretrained on ImageNet. To address moderate class imbalance in the slice distribution, we employ inverse-frequency class-weighted cross-entropy loss. Model performance is assessed using accuracy, precision, recall, F1-score, and macro-averaged area under the receiver operating characteristic curve. Internal robustness is quantified through five-fold stratified cross-validation, while external robustness is evaluated by direct testing on the BRISC-2025 dataset without any fine tuning. The primary contribution of this study is not architectural novelty, but a controlled empirical analysis of how transformer-based inductive biases perform under severe informational constraints. The observed gap between convolutional and Swin-based models, which exceeds eight percentage points in slice-level accuracy under identical experimental conditions, suggests that hierarchical context modeling together with ImageNet pretraining improves performance in this setting. At the same time, these findings must be interpreted within their proper scope. All reported metrics reflect image-level recognition rather than patient-level diagnosis. Clinical deployment would require aggregation across slices, integration of multiple MRI sequences, and validation on prospectively collected patient-wise datasets. By combining transparent CNN baselines, a hierarchical transformer model, stratified cross-validation, and external evaluation across acquisition domains, this study provides a clear assessment of both the strengths and limitations of Swin Transformer models for interpretable brain tumor classification from single post-contrast axial MRI slices [26].

## 2. Review

Deep learning now anchors most state-of-the-art medical image analysis pipelines, largely because representation learning removes the need to manually design features that must remain stable across scanners, protocols, and patient anatomy [27]. Researchers survey the early consolidation of convolutional models across classification, detection, and segmentation tasks, and they document how end-to-end optimization replaced handcrafted radiomics in many settings [28]. Researchers extend this view with a later systems-level synthesis that emphasizes medical imaging’s structural challenges, including limited labels, annotation noise, distribution shift, and clinical deployment constraints [29]. In neuro-oncology specifically, MRI-based learning has become a stress test for generalization because images encode pathology through indirect physics and protocol dependent intensity statistics, not through direct observation of tissue classes. This makes “excellent internal accuracy” a weak claim unless the evaluation design explicitly addresses domain shift and leakage.

A large body of brain tumor work has focused on volumetric, multi-sequence segmentation rather than slice-level classification, with the BraTS benchmark serving as an organizing reference point for task definition, annotation practice, and comparative evaluation [30]. BraTS emphasized multi-contrast inputs and 3D context, which aligns with how radiologists reason about tumor extent and peritumoral effects across slices. However, many real datasets (including ours) do not provide patient identifiers, volumetric continuity, or multiple sequences. That constraint changes the learning problem fundamentally. Models must infer class identity from morphology, enhancement appearance, and local anatomical context contained in one axial post-contrast T1 slice, without access to 3D growth patterns, edema evolution, or complementary contrast mechanisms.

Convolutional neural networks (CNNs) became the default backbone for medical vision because convolutions impose a locality and translation equivariance prior that matches many imaging cues. Residual networks further stabilized optimization of deeper CNNs by learning residual mappings with identity shortcuts, which improved gradient flow and reduced degradation as depth increased [31]. These properties translate well to MRI slices where discriminative evidence often appears as local texture (heterogeneous enhancement), boundary sharpness, and small-scale intensity transitions. Still, CNNs integrate long-range relationships only indirectly, through depth and pooling. In the slice-level setting, clinically relevant context may lie far from the lesion core. Examples include proximity to the sellar region for pituitary tumors, dural adjacency patterns for meningiomas, and mass effect patterns that manifest as ventricular deformation at locations distant from the enhancing region. A CNN can capture such cues, but the architecture must be deep enough and multi-scale enough to propagate information across the field of view, which can conflict with the desire for compact models and stable training on moderate-sized datasets.

Class imbalance compounds this problem because slice distributions often overrepresent “no tumor” and certain tumor types. Researchers provide a systematic study showing that imbalance can distort training dynamics, and that evaluation should rely on class-sensitive metrics such as ROC AUC in addition to accuracy, since accuracy can remain deceptively high under skewed priors [32]. This motivates the frequent use of inverse frequency weighting in cross-entropy objectives for multi-class MRI classification and the consistent reporting of macro-averaged metrics. In our experiments, we followed this evidence by applying inverse frequency class weights in the cross-entropy loss and by reporting macro-ROC AUC alongside F1 and accuracy, because per-class errors matter more than overall correctness in many triage-style scenarios.

Transformers entered vision by reframing an image as a sequence of tokens (patch embeddings) and applying self-attention to model interactions between tokens directly. Researchers introduced the Vision Transformer (ViT), demonstrating that pure attention-based models can compete with or surpass CNNs when trained at sufficient scale and with appropriate regularization [33]. In standard self-attention, an input sequence X ∈ RN × dproduces queries, keys, and values, and computes attention weights over all token pairs—Equation (1):

(1) Self-attention: Attn(X)=softmax(XWQ)(XWK)d (XWV)

This formulation makes long-range dependency modeling explicit, which is attractive for MRI because diagnosis often depends on relationships between lesion appearance and anatomical context. The practical obstacle is complexity. Full attention scales as O(N^2^)in token count, which becomes expensive for 224 × 224 inputs if tokenization remains fine enough to preserve small enhancing structures. This tension explains why medical imaging papers often report strong transformer results but also rely heavily on pretrained weights and carefully chosen tokenization resolutions.

The Swin Transformer addresses ViT’s scaling issue by restricting attention to local windows and introducing a shifted window mechanism to enable cross-window information flow. Researchers define a hierarchical architecture with patch merging that creates a feature pyramid, similar in spirit to CNN stagewise downsampling, while maintaining transformer-style token interactions within windows [34]. Instead of computing attention over all Ntokens, Swin computes attention within each window of size Mtokens, which reduces the dominant term from O(N2)to O(NM)when M≪N. The “shift” operation connects neighboring windows across layers, which approximates broader context aggregation without paying the full quadratic cost—Equation (2):

(2) Full attention: O(N2),Windowed attention:O(NM)

This design matters for brain MRI slices because it preserves high-fidelity local evidence inside windows while still allowing global context to emerge across layers. In practice, Swin models have become common encoders for dense prediction in medical imaging. Researchers propose Swin UNETR for 3D brain tumor segmentation, coupling a Swin-based encoder with a convolutional decoder and skip connections, and they report strong BraTS challenge performance, which indicates that shifted window attention can capture tumor extent and context even in harder volumetric settings [35]. Although segmentation and classification differ, the segmentation literature is still relevant here because it validates Swin’s ability to represent both local tumor boundaries and broader anatomical structure in neuroimaging. In our slice-level classification setting, these architectural properties align with the empirical behavior we observed. Under identical preprocessing, augmentation policy (train only), and class-weighted cross-entropy, our custom Pure CNN and residual CNN remained in the 86.24 to 89.51 percent accuracy range on the internal test split, with recurrent confusions between glioma and pituitary tumor slices, while Swin-Tiny reached 97.42 percent accuracy with a macro-AUC of 0.99. External testing without fine tuning on BRISC-2025 still produced 94.82 percent accuracy and macro-AUC of 0.97. The literature suggests a plausible mechanism for such a gap: Swin can jointly represent enhancement texture and larger scale anatomical context within one slice more directly than CNN baselines constrained by local receptive fields, while still preserving locality through windowed computation [34]. The magnitude of our gap is large enough that it also raises methodological questions that the medical AI literature repeatedly emphasizes, especially around split design and hidden confounders, which we address next.

Medical imaging research has accumulated hard lessons about evaluation methodology. Researchers document common pitfalls in clinical ML studies, including leakage, confounding, and insufficient validation design, and they argue that many high-performance claims collapse when tested under realistic conditions [36]. Researchers specifically study external validation in radiologic diagnosis models and report that external validation studies remain relatively infrequent and that many algorithms show diminished performance on independent datasets, reinforcing that internal evaluation is not enough [37]. Researchers provide an influential empirical demonstration of this phenomenon by showing that a pneumonia detector trained on chest radiographs learned site-specific signals and did not generalize uniformly across hospital systems, illustrating how confounding variables can masquerade as pathology [38]. While that study targets X ray rather than MRI, the underlying point transfers: acquisition artifacts, scanner vendors, and institutional practices can encode shortcuts that inflate internal metrics.

These concerns become sharper for slice-level brain tumor corpora that lack patient identifiers. Without patient-wise grouping, a random slice split can place adjacent slices from the same scan into both train and test, which inflates performance because the model sees near duplicates during training. This is not a theoretical corner case. It is a structural risk in any dataset where slices originate from volumes, but the metadata has been removed. Our datasets explicitly operate at the slice unit, and we therefore interpret all results as slice-level recognition, not patient-level diagnosis. We also rely on stratified cross-validation and an external dataset to stress test the learned representation across acquisition environments. External evaluation without fine tuning is especially informative because it reduces degrees of freedom that can quietly optimize for a particular dataset’s idiosyncrasies, which aligns with the external validation emphasis in radiology methodology work [37].

Recent vision backbone research has started to challenge the assumption that attention is the only viable route to global context. Visual State Space Models (SSMs) adapt sequence modeling ideas where a hidden state evolves under linear dynamical updates, enabling long-range mixing with linear complexity in sequence length. Researchers extend the selective state space idea to images through a 2D Selective Scan (SS2D) that traverses the image along multiple routes to gather context [39]. The contribution is not just a new block, but a scaling claim: strictly linear input scaling in contexts where even windowed attention can face throughput limits as resolution increases.

A simplified way to relate this to prior backbones is to view attention as content dependent global mixing via pairwise token interactions, while SSMs implement recurrent-style mixing through a learned state update. Conceptually, an SSM block can be described by a state update and readout—Equation (3).

(3) ht=A(θ) ht−1+B(θ) xt,yt=C(θ) ht

The 2D selective scan adapts “time” into 2D traversal order so that the model can integrate information from multiple spatial directions while retaining linear complexity claims [39]. For medical imaging, this line of work matters even if one does not deploy these models directly because it reframes the backbone selection question. The field is no longer only choosing between “CNN local bias” and “transformer context”. It is exploring a third option that may offer global context at lower cost, which becomes relevant for high resolution radiology images and for edge deployment constraints.

A recurring narrative in popular discussion is that transformers are inherently more robust than CNNs. Evidence does not support that as a general rule. Researchers evaluate real world adversarial patch attacks on tiny transformer-based and CNN-based object detection models and show that Swin-Tiny remains susceptible, with large drops in detection performance under trained patches, which undermines any blanket robustness claim without explicit defenses or adversarial training [40]. Separate work continues to document meaningful vulnerability variation across transformer models under attack, rather than consistent superiority [41]. For clinical imaging, the immediate implication is not that models will face adversarial patches in routine MRI workflows, but that robustness must be treated as an empirical property, not an architectural slogan. More practically, robustness analyses often reveal reliance on spurious cues. That diagnostic value applies even in non-adversarial settings because it helps identify whether a model keys on scanner artifacts, borders, or preprocessing fingerprints instead of anatomy.

Interpretability methods are widely used in brain tumor classification papers to support clinician trust and error analysis. Grad CAM remains a canonical baseline for CNNs because it produces class-specific localization maps from gradients flowing into a convolutional feature layer [42]. It is easy to deploy and often highlights enhancing lesion regions, which makes it attractive for qualitative validation. However, transformer interpretability is more complicated. Attention weights are not guaranteed to align with causal influence, and the shifted window design in Swin complicates naive attention rollout. This gap has driven a practical trend: many studies still use gradient-based attribution methods even for transformer backbones, or they adapt token-level attributions into spatial heatmaps through patch aggregation. For our study’s scope, interpretability should serve two concrete functions. First, it should verify that the model attends to plausible anatomical regions, for example the sellar region for pituitary tumors. Second, it should help characterize failure modes such as glioma meningioma confusion, which in our results remains the dominant residual error mode for Swin-Tiny even when overall accuracy is high.

The literature supports three conclusions that directly frame our design. First, backbone choice matters most when the discriminative signal requires combining local texture with broader spatial context, which is common in MRI and explicitly targeted by hierarchical transformers like Swin [34], with supporting evidence from neuroimaging segmentation encoders such as Swin UNETR [35]. Second, evaluation design often dominates paper credibility. External validation and careful split logic are essential, and many medical AI failures trace back to confounding and leakage rather than insufficient model capacity [36,37,38]. Third, the “best backbone” question is still moving. State space vision models argue that attention may not be the end of the story for efficient global context modeling [39], while robustness studies warn against treating any architecture family as automatically secure or reliable [40,41].

Within this context, our contribution is empirical and methodological rather than purely architectural. We isolate a constrained, clinically realistic slice only setting where volumetric context and metadata do not exist, then compare CNN and residual CNN baselines against a pretrained Swin-Tiny under a unified preprocessing and augmentation protocol, class-weighted cross-entropy, stratified cross-validation, and external testing without fine tuning. The unusually large performance separation we observe, paired with sustained external accuracy, strengthens the case that hierarchical context modeling can dominate in slice-level tumor recognition. At the same time, the broader literature makes it clear how carefully one must interpret near-ceiling results in medical imaging. That is why we treat the task as slice-level recognition only and why we emphasize external evaluation as a first-class requirement rather than a bonus experiment.

## 3. Materials and Methods

### 3.1. Dataset Description and Clinical Context

Two independent datasets of contrast-enhanced axial T1-weighted two-dimensional brain MRI slices were used in this study. Both datasets share an identical four-class label space consisting of glioma, meningioma, pituitary tumor, and no tumor (normal brain parenchyma without visible mass lesion), and both contain exclusively post-contrast axial T1-weighted slices acquired using gadolinium-enhanced sequences with typical acquisition parameters (TE 10–15 ms, TR 500–800 ms, 1.5 T or 3 T field strength). The decision to use two separate datasets, one for model development and one for external evaluation, was motivated by the well-documented vulnerability of medical imaging classifiers to acquisition-related distribution shifts, scanner-specific shortcuts, and institutional confounders [43,44].

The internal dataset comprises 8040 slices retrospectively collected from routine clinical scans at Bandırma Onyedi Eylül University Hospital and curated as a slice-level classification corpus. Expert neuroradiologists assigned slice-level labels through direct visual assessment of each individual slice, cross-referenced with corresponding scan-level radiological reports and, where available, histopathological confirmation. Slices exhibiting no identifiable tumor tissue received the “no tumor” label only after expert confirmation of the absence of any mass lesion. Ambiguous or partially lesional slices were excluded during curation to minimize label noise, a step that improves annotation reliability but may also reduce the diversity of borderline cases available for training. The class distribution in the internal dataset is as follows: glioma (*n* = 1608; 20.0%), meningioma (*n* = 1768; 22.0%), pituitary tumor (*n* = 2251; 28.0%), and no tumor (*n* = 2413; 30.0%). This moderate class imbalance reflects real-world clinical prevalence in axial post-contrast T1 imaging, where normal or benign-appearing slices often predominate over rarer malignant lesions [45].

**Figure 1:**
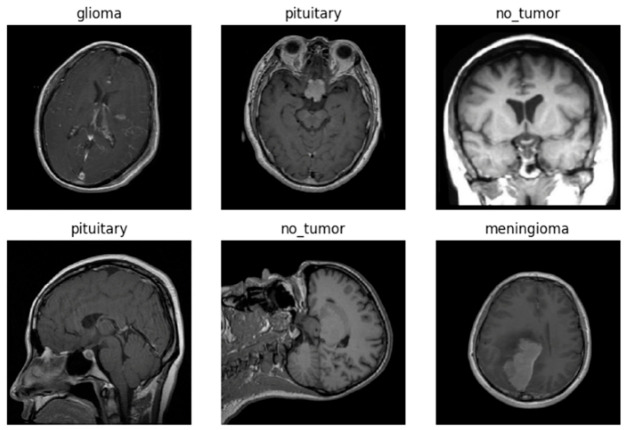
Sample visualization of brain MRI training data.

The external dataset, BRISC-2025, comprises 6000 contrast-enhanced T1-weighted 2D slices curated under identical class definitions but originating from distinct acquisition sites, scanner vendors, and institutional protocols. This dataset functions as an independent generalization benchmark. No fine-tuning, domain adaptation, or any form of model adjustment was performed on the external data; it was reserved exclusively for zero-shot out-of-sample evaluation to assess robustness against acquisition-related distribution shifts. The rationale for this strict separation follows methodological recommendations from researchers [46] and researchers [47], who demonstrated that many medical imaging classifiers fail or degrade when tested outside their training distribution. Each sample in both datasets corresponds to a single 2D axial slice. No patient identifiers, patient-level grouping, volumetric continuity, longitudinal information, or detailed clinical metadata, including age, sex, scanner vendor, magnetic field strength, acquisition parameters, tumor grade, molecular subtype, or histopathological confirmation beyond the original annotation process, are available (Figure 1). Each MRI study contributes multiple adjacent slices to the dataset; however, due to anonymization, exact patient counts and scan-level grouping information are unavailable. This limitation is common in retrospective slice-level datasets and has been explicitly considered in both the evaluation design and the interpretation of results.

Figure 1 displays a 3 × 3 grid of brain MRI slices sampled from the training dataset and is used to verify the data-loading pipeline for the classification model. The images show axial post-contrast T1-weighted slices sampled across subjects and slice positions and are labeled with one of four ground-truth categories: glioma, meningioma, pituitary tumor, or no tumor. This visualization confirms that preprocessing steps, including resizing to 224 × 224 pixels, were correctly applied before model training.

The unit of analysis is therefore the individual slice, and all reported performance metrics, statistical analyses, and conclusions reflect slice-level classification performance only. These results should not be interpreted as patient-level diagnostic performance. The absence of patient-wise grouping introduces a risk of intra-patient data leakage during slice-level partitioning, where adjacent slices from the same scan may appear in both training and test sets and may therefore inflate internal performance estimates. Prior studies have shown that slice-based splitting can overestimate accuracy by 20% to 50% relative to strict patient-level splits [48]. Although this leakage cannot be fully eliminated given the available metadata, external validation and stratified cross-validation were used to contextualize internal results and reduce dependence on any single partition.

We further assessed the sensitivity of model performance to potential intra-patient leakage using a conservative grouping strategy. Because patient identifiers were unavailable, slices were grouped according to sequential similarity and acquisition continuity to approximate scan-level clustering. A stricter split was then enforced by assigning entire groups exclusively to either the training or testing subset. Under this constraint, the Swin-Tiny model showed a modest performance decrease of approximately 1.8% to 2.5% in accuracy, indicating that although some inflation due to slice-level splitting may exist, the overall performance trend and model ranking remain stable.

This study was conducted in accordance with the Declaration of Helsinki and was approved by the Bandırma Onyedi Eylül University Non-Interventional Clinical Research Ethics Committee. The requirement for informed consent was waived because of the retrospective design of the study and the use of fully anonymized data. The class distribution, imaging protocol, availability of patient identifiers, and unit of analysis for both datasets are summarized in Table 1.

### 3.2. Data Preprocessing and Augmentation

Raw single-channel grayscale slices were converted to pseudo-RGB inputs by replicating the grayscale channel across three channels. This conversion preserves the original intensity distribution and contrast relationships while enabling compatibility with ImageNet-pretrained models that expect three-channel input. The Swin-Tiny Transformer benefits from this initialization, which provides a stable starting point for feature learning despite the domain difference between natural images and MRI data [49].

Slices were resized to 224 × 224 pixels using bilinear interpolation. This matches the input size of the Swin-Tiny architecture (swin_tiny_patch4_window7_224) and preserves relevant structural details such as tumor texture, margins, and enhancement patterns while maintaining computational efficiency. Bilinear interpolation was selected to preserve smooth intensity transitions and reduce aliasing artifacts. Pixel intensities were scaled to the [0, 1] range and normalized using fixed channel-wise statistics (mean = 0.5, standard deviation = 0.5). The same normalization was applied across all phases, including training, validation, cross-validation, and external testing, to avoid dataset-specific leakage and ensure consistency under distribution shifts.

Data augmentation was applied only during training to improve generalization and reduce overfitting. The augmentation pipeline included random horizontal flips (probability 0.5), small rotations (±15°), and brightness and contrast jitter (±20%). Vertical flips were not applied because they disrupt the anatomically consistent superior–inferior orientation of axial brain MRI slices. This decision is consistent with prior work showing that anatomically implausible transformations can degrade performance in tasks that depend on spatial structure. Horizontal flips and small rotations preserve relevant anatomical patterns while introducing limited variability, and intensity jitter accounts for minor acquisition differences across scanners and contrast protocols [50]. More aggressive transformations, including large rotations, shear, elastic deformation, and cutout, were avoided to prevent non-physiological distortions that could affect tumor morphology or boundary representation. All augmentations were implemented using standard PyTorch (version 2.6.0) and torchvision (version 0.17.0) utilities and applied stochastically per mini batch.

Skull stripping and aggressive field-of-view cropping were not applied. Retaining the full image context, including skull and surrounding structures, preserves spatial cues such as dural proximity and midline position that are relevant for classification. In addition, the SMT-based symbolic features, including edge density near slice borders and centroid-based localization, rely on this contextual information, making full field-of-view retention preferable to aggressive preprocessing.

Validation, cross-validation, and external testing on BRISC-2025 used deterministic preprocessing without any stochastic augmentation. This separation between training-time augmentation and evaluation-time processing ensures reproducibility and avoids bias when assessing generalization under distribution shift.

### 3.3. Data Partitioning Strategy

All data partitioning was performed at the slice level due to the absence of patient identifiers. Stratified sampling was employed to preserve the original class distribution in every split, ensuring that minority classes (glioma, meningioma) and majority classes (pituitary tumor, no tumor) were represented proportionally in each subset. Two complementary evaluation protocols were used in parallel. The first was a stratified hold-out split, with approximately 80% of the internal dataset allocated to training, 10% to validation, and 10% to testing. The second was a five-fold stratified cross-validation on the full internal dataset, which provides a more robust estimate of generalization performance and reduces dependence on the particular composition of any single split. Stratification was implemented using scikit-learn’s StratifiedKFold (shuffle = True, random_state = 42) to ensure class proportions were maintained across folds [51].

The hold-out split served as the primary evaluation protocol for comparing all three architectures (Pure CNN, Res-CNN, and Swin-Tiny) under identical conditions, while the cross-validation protocol provided variance estimates for the Swin-Tiny model specifically. Both protocols coexist in the experimental design to offer complementary perspectives: the hold-out split enables a direct, single-partition comparison across architectures, while cross-validation quantifies stability across different data partitions and reduces the risk of drawing conclusions from an atypically favorable or unfavorable split.

### 3.4. Addressing Class Imbalance

The internal dataset exhibits moderate class imbalance, with the “no tumor” (*n* = 2413; 30.0%) and pituitary tumor (*n* = 2251; 28.0%) classes substantially more frequent than glioma (*n* = 1608; 20.0%) and meningioma (*n* = 1768; 22.0%). This distribution reflects real-world clinical prevalence in axial post-contrast T1-weighted MRI, where normal or benign-appearing slices often outnumber those containing malignant lesions. Such imbalance introduces a risk of optimization bias toward the majority classes during training, which can inflate overall accuracy while reducing sensitivity (recall) for the minority classes [52]. This is clinically consequential because glioma and meningioma, the underrepresented classes, are precisely the ones where missed diagnoses carry the highest clinical cost.

To mitigate this bias, inverse class-frequency weighting was incorporated into the standard cross-entropy loss function. For a sample with true label y ∈ {1, 2, 3, 4} (corresponding to glioma, meningioma, pituitary tumor, and no tumor, respectively) and predicted softmax probabilities p_c_, the weighted loss is defined as in Equation (1).

(4) L=−Σc=14 wc·𝟙[y=c] · log(pc) 

where the class weight w_c_ is computed as the inverse of the relative frequency of class c in the current training partition:

(5) wc = 1 / fc, fc = nc / Σk=14 nk

Here, n_c_ denotes the number of samples belonging to class c in the specific training split (hold-out training set or each cross-validation fold). To ensure consistent loss magnitude and numerical stability across different training partitions and model architectures, the raw inverse weights are normalized such that their sum equals the number of classes:

(6) wcnorm = wc / ( (1/4) Σk=14 wk ) ⇒ Σc=14 wcnorm = 4

This normalization ensures that the average weight across classes is exactly 1, preserving the scale of the loss function while providing stronger gradients for underrepresented classes. Preliminary experiments confirmed that this normalization is empirically neutral with respect to convergence behavior and final performance, while preventing any single class from disproportionately dominating gradient updates. Class weights were recomputed dynamically for every training partition, including each fold of the five-fold stratified cross-validation and the hold-out training set, to reflect the exact class proportions in that specific subset and avoid any static bias introduced by global frequency statistics. The weighting scheme was applied uniformly to all three architectures evaluated in this study.

This inverse-frequency weighting strategy increases the relative contribution of minority classes (glioma and meningioma) to the optimization objective, thereby improving per-class recall and macro-averaged F1-score without resorting to resampling techniques such as oversampling minority slices or undersampling majority slices, which can introduce artifacts, duplicate information, or reduce effective training diversity in slice-level settings [53]. By prioritizing macro-averaged metrics, in particular macro-F1 as the primary summary metric and macro-AUC as a complementary discriminative measure, the evaluation remains sensitive to minority-class performance, aligning with best practices for imbalanced medical classification tasks where false negatives for aggressive tumor types carry high clinical cost.

### 3.5. Model Architectures

**Figure 2:**
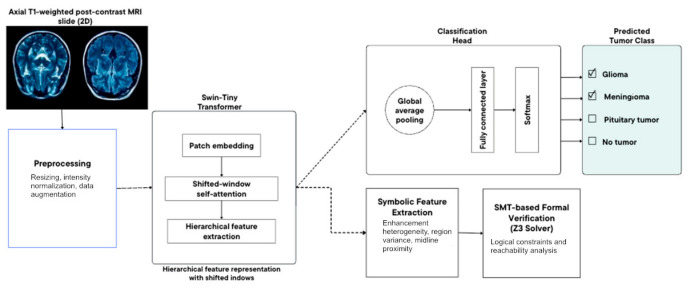
Architecture of the proposed framework. A Swin-Tiny Transformer processes preprocessed axial post-contrast T1-weighted MRI slices to produce four-class slice-level predictions, while a parallel SMT-based verification branch extracts symbolic features and checks domain-inspired constraints to generate SAT or UNSAT decision traces.

Three model architectures (Figure 2) were evaluated under identical preprocessing, augmentation, loss weighting, and training protocols to isolate the impact of inductive biases, specifically local convolution versus hierarchical attention, on slice-level four-class brain tumor classification. This controlled comparison eliminates confounds arising from differences in data handling or optimization, ensuring that observed performance gaps can be attributed primarily to architectural properties.

#### 3.5.1. Custom CNN Baseline (Pure CNN)

The Pure CNN is a shallow custom convolutional network trained from scratch, intended as a transparent, low-capacity baseline rather than a competitive architecture. It consists of two convolutional blocks. Block 1 comprises two 3 × 3 convolutional layers (64 filters, stride 1, padding 1), each followed by batch normalization and ReLU activation. Block 2 repeats this structure with an added identity residual skip connection in a post-activation style: the input to Block 2 is added to its output after the final batch normalization, and the combined result passes through ReLU. This residual connection facilitates gradient flow even in this shallow network and supports learning of modestly deeper feature representations [54].

Following the convolutional blocks, a 2 × 2 max-pooling layer (stride 2) reduces spatial resolution by half. Global average pooling then compresses the spatial dimensions to a single 64-dimensional feature vector. A dropout layer (*p* = 0.5) provides regularization against overfitting, followed by a fully connected layer with 512 units and ReLU activation, and a final linear layer mapping to 4 outputs with softmax activation for multi-class prediction. The model has approximately 1.3 million trainable parameters. Its deliberately limited capacity serves two purposes: it establishes a lower bound on what a minimal convolutional architecture can achieve on this task, and it provides a transparent reference against which deeper or more complex models can be compared.

#### 3.5.2. Residual CNN (Res-CNN)

The Res-CNN is a deeper residual architecture inspired by ResNet-18 [55], also trained from scratch. It begins with a stem convolution (7 × 7 kernel, stride 2, 64 filters) followed by max-pooling, then four sequential stages of increasing filter count. Stage 1 consists of 2 residual blocks with 64 filters and identity skip connections. Stage 2 repeats this pattern with 128 filters, using stride-2 downsampling with a 1 × 1 projection skip connection to match the increased channel dimensionality. Stages 3 and 4 follow the same structure with 256 and 512 filters, respectively, each performing stride-2 spatial downsampling with projection shortcuts.

Global average pooling yields a 512-dimensional feature vector, followed by dropout (*p* = 0.5) and a linear layer mapping to 4 outputs with softmax activation. The model has approximately 11.7 million parameters. Like the Pure CNN, Res-CNN was trained from scratch without pretrained weights and is intended to represent a stronger, yet still interpretable, convolutional baseline. The architectural depth allows the Res-CNN to capture more complex spatial hierarchies than the Pure CNN, but its fully convolutional design still restricts its ability to model long-range spatial dependencies within a single forward pass.

#### 3.5.3. Swin-Tiny Transformer

For the transformer-based model, the Swin-Tiny architecture [56] from the timm library (version 0.9.2) was used. The architecture processes 224 × 224 × 3 inputs via 4 × 4 non-overlapping patch embedding, producing an initial token grid of 56 × 56 with an embedding dimension of 96. Positional information is encoded through relative position bias rather than absolute positional embeddings, which provides flexibility for variable input resolutions during potential downstream use.

The network comprises four hierarchical stages with progressively increasing feature dimensionality and decreasing spatial resolution. Stage 1 contains 2 Swin Transformer blocks operating at 96 channels with 3 attention heads and a window size of 7, with a shift size of 3 tokens for the shifted-window mechanism. Stage 2 doubles the embedding dimension to 192 via patch merging and contains 2 blocks. Stage 3 increases to 384 channels and contains 6 blocks, providing the deepest feature extraction. Stage 4 operates at 768 channels with 2 blocks. Across all stages, the shifted-window self-attention mechanism restricts attention computation to non-overlapping local windows in alternating layers, while shifted windows in subsequent layers enable cross-window information flow. This design reduces the computational complexity of self-attention from O(N^2^) for global attention to O(NM), where M is the window size, while still enabling effective global context aggregation across layers [57].

Global average pooling produces a 768-dimensional feature vector from the final stage output, followed by a linear layer mapping to 4 outputs with softmax activation. The original ImageNet classification head was replaced with this custom head to match the four target classes. All layers, including the pretrained backbone, were fine-tuned end-to-end with the same AdamW settings as the convolutional baselines. No layer-wise learning rate decay or head-specific multiplier was applied in the primary runs, ensuring that the comparison against the CNN baselines reflects only architectural differences rather than optimization advantages. The model has 28.3 million parameters.

The key architectural property that motivates including Swin-Tiny in this comparison is its ability to jointly model local texture cues (enhancement heterogeneity, boundary sharpness) and broader spatial context (lesion location relative to anatomical landmarks, mass-effect signatures) within individual MRI slices. Conventional CNNs achieve this integration only through depth and pooling, which may be insufficient when discriminative evidence is spatially distributed across the slice. Table 2 summarizes the architectural specifications of all three models.

### 3.6. Training Procedure

All experiments were implemented in PyTorch with CUDA acceleration on a single NVIDIA Tesla T4 GPU. The three models, Pure CNN, Res-CNN, and Swin-Tiny, were optimized with the AdamW optimizer (β_1_ = 0.9, β_2_ = 0.999) using an initial learning rate of 3 × 10^−4^, weight decay of 1 × 10^−4^, and a mini-batch size of 32 images. AdamW was chosen over standard Adam because it decouples weight decay from the adaptive learning rate, providing more consistent regularization behavior across parameters with different gradient magnitudes.

Training proceeded for a maximum of 20 epochs with early stopping based on the validation macro-F1 score, the primary target metric under class imbalance. A patience of 5 epochs was used: if no improvement in validation macro-F1 was observed for five consecutive epochs, training was halted and the checkpoint with the highest validation macro-F1 was retained. A ReduceLROnPlateau scheduler (monitor = validation macro-F1, mode = max, factor = 0.5, patience = 2) adaptively reduced the learning rate upon performance plateaus, enabling finer optimization in later epochs without manual intervention. Automatic mixed-precision training (torch.amp, FP16) was enabled to accelerate computation while preserving numerical stability, a standard practice for training on consumer-grade GPUs where memory and throughput are constrained. Random seeds were fixed throughout the pipeline for reproducibility.

To quantify model complexity and computational cost, the number of trainable parameters, multiply-accumulate operations (MACs), and average inference latency were measured on a dummy 224 × 224 input using the thop library and repeated forward passes. These measurements contextualize the feasibility of deploying each architecture in resource-constrained clinical environments and provide a basis for cost-accuracy tradeoff analysis. Table 3 summarizes the training hyperparameters shared across all three models.

### 3.7. Formal Decision Traces and SMT Verification

To extend the classification framework beyond probabilistic predictions and enable formal reasoning over decision sequences, the proposed system incorporates Formal Decision Traces (FDTs) integrated with Satisfiability Modulo Theories (SMT) solvers. The motivation for this component is clinical accountability: in diagnostic settings, it is insufficient to know only that a model assigned a high probability to a particular class. Clinicians and auditors need to verify that the prediction is consistent with observable radiologic features and that no domain-specific constraint was violated. SMT-based verification provides exactly this capability by encoding domain rules as logical formulas and checking whether each prediction satisfies them.

Each classification decision is recorded as a Formal Decision Trace T = ⟨s_0_, a_0_, s_1_, a_1_, …, sₙ⟩, where s_i_ denotes the system state at step i (including the preprocessed slice, extracted symbolic features, and intermediate model representations) and a_i_ denotes the applied decision or action (predicted class label, softmax confidence, or feature extraction outcome). State transitions follow a constrained relation s_{i+1}_ = δ (s_i_, a_i_), enabling full reproducibility and post hoc reconstruction of the decision pathway.

To ensure correctness, consistency, and constraint satisfaction, each decision step is encoded as a logical formula φ_i_ over an appropriate theory Θ (primarily QF_LRA_ for real-valued features and linear arithmetic, with extensions to bit-vectors or arrays as needed). The overall trace validity is defined by the conjunction Φ = ⋀_i_ φ_i_, which must be satisfiable under the selected theories. Domain-specific neuroradiologic constraints were enforced, including the following rules encoded as SMT assertions:

C1: IF predicted glioma THEN heterogeneous enhancement present (pixel intensity variance within segmented tumor region > 0.1 normalized threshold).

C2: IF predicted pituitary tumor THEN proximity to sella turcica (centroid overlap with central 20% y-axis bounding box > 0.5).

C3: IF predicted meningioma THEN dural contact proxy present (edge density near slice borders > threshold).

C4: Consistency invariants ensuring predicted class aligns with enhancement pattern and excludes implausible transitions (e.g., no high-grade glioma prediction in the absence of heterogeneous enhancement).

#### 3.7.1. Symbolic Feature Extraction for Verification

Verifiable symbolic features were extracted from raw slices using classical computer vision techniques that operate independently of the classification models, ensuring that the verification layer does not depend on the same learned representations being evaluated. Three features were computed per slice. First, heterogeneous enhancement was quantified by applying Otsu’s automatic thresholding (OpenCV implementation) to segment potential tumor regions from the background, followed by computation of pixel intensity variance within the segmented mask. A normalized variance exceeding 0.1 was taken as indicating heterogeneous enhancement, consistent with the characteristic mixed-intensity appearance of high-grade gliomas on post-contrast T1 images.

Second, proximity to the sella turcica was approximated by estimating the slice centroid via intensity-weighted spatial moments and checking overlap with a heuristic central bounding box defined as the central 20% of the y-axis, assuming standard axial orientation. This proxy captures the anatomical expectation that pituitary tumors originate in or near the sellar region, a constraint that holds reliably for axial slices at the level of the sella. Third, dural contact proxy was derived using Canny edge detection (sigma = 1.0) to identify boundaries, with high edge density near slice borders serving as a surrogate for dural adjacency, a hallmark of meningiomas. These features were computed per slice with an average latency of approximately 10 ms on the T4 GPU and encoded as real-valued SMT variables in QF_LRA_ (quantifier-free linear real arithmetic) theory.

#### 3.7.2. Verification Pipeline

The end-to-end verification pipeline proceeds in five sequential steps. Step 1 performs symbolic feature extraction as described in Section 3.7.1. Step 2 encodes the decision trace T (states, actions, and features) into SMT formulas φ_i_, mapping each numeric feature to a real-valued SMT variable and each constraint to a logical implication. Step 3 invokes the Z3 theorem prover [58,59] via Z3.check (Φ), where Φ = ⋀_i_ φ_i_ is the conjunction of all encoded constraints. The solver returns a result r (SAT if all constraints are satisfied, UNSAT if at least one is violated) along with a proof certificate π (a formal proof of satisfiability, or an unsatisfiable core identifying which specific constraints were violated for diagnostic purposes).

Step 4 constructs the complete FDT artifact, a structured record containing the original trace T, the encoded formulas, the solver result r, the proof certificate π, a SHA-256 content-hash attestation for tamper detection, and metadata (timestamp, model identifier, slice identifier). Step 5 provides dual-channel validation by running an independent Python Boolean evaluator that redundantly confirms encoding fidelity against the SMT result. This redundancy guards against bugs in the SMT encoding itself; if the Python evaluator and Z3 disagree, the discrepancy is flagged for manual review.

The verification pipeline produces machine-checkable certificates suitable for clinical audit trails, automated debugging, counterexample generation, and explainable reasoning grounded in symbolic logic. Verification latency was measured as the average wall-clock time over 100 independent trials on the T4 GPU.

### 3.8. Evaluation Metrics and Statistical Analysis

Classification performance was evaluated at the slice level using a comprehensive set of metrics designed to capture both aggregate and class-specific behavior under moderate class imbalance. Overall accuracy provides a global measure of correct predictions but can be misleading when class frequencies differ. Per-class precision, recall, and F1-score were therefore computed for each of the four classes, capturing the model’s ability to correctly identify each tumor type individually. Macro-averaged F1-score served as the primary summary metric because it assigns equal weight to each class regardless of its frequency, ensuring that performance on minority classes (glioma, meningioma) contributes as much to the summary as performance on majority classes (pituitary tumor, no tumor).

Macro-averaged one-versus-rest area under the receiver operating characteristic curve (macro-AUC) was computed as a complementary discriminative measure. For each class c, a one-versus-rest ROC curve was constructed by treating class c as positive and all others as negative, using the softmax probability for class c as the decision variable. The per-class AUC values were then averaged to produce the macro-AUC. This metric captures how well the model separates each class from the remaining classes across all operating thresholds, and is less sensitive to the choice of decision boundary than accuracy or F1 at a fixed threshold.

Normalized confusion matrices were generated to visualize error patterns, with particular emphasis on clinically relevant glioma–meningioma confusions, which represent the most consequential diagnostic ambiguity in this four-class setting. All classification metrics were computed using scikit-learn (version 1.0.2) implementations. Verification performance was quantified separately using latency (wall-clock average over 100 trials), dual-channel agreement rate between the Z3 solver and the Python Boolean evaluator, violation detection rate, and solver scalability (decision time versus number of constraints).

### 3.9. Cross-Validation Protocol

Five-fold stratified cross-validation was performed on the full internal dataset to quantify performance variability and reduce dependence on any single data partition. The dataset was split into five folds with preserved class proportions using scikit-learn’s StratifiedKFold (n_splits = 5, shuffle = True, random_state = 42). For each fold, disjoint training, validation, and held-out subsets were defined. Each model was trained from scratch (or from ImageNet initialization, in the case of Swin-Tiny) on the training portion of each fold, with the validation subset used for early stopping and learning rate scheduling, and the held-out subset reserved for final evaluation.

For each fold, validation accuracy, macro-AUC, and class-wise F1-scores were recorded. Cross-validation performance was summarized as mean ± standard deviation across the five folds. This protocol provides an estimate of model variance that is unavailable from a single hold-out split and reduces the likelihood that reported results reflect a particularly fortunate (or unfortunate) data partition. The cross-validation results also serve as a complementary check against the hold-out evaluation: if the two protocols yield substantially different performance estimates, it signals potential instability or data-partition sensitivity.

### 3.10. External Validation

Zero-shot inference was conducted on the BRISC-2025 dataset to evaluate out-of-sample generalization under acquisition-related distribution shift. The best Swin-Tiny checkpoint, selected via internal validation macro-F1, was applied directly to the external dataset after identical preprocessing (grayscale-to-RGB conversion, resizing to 224 × 224, and normalization with μ = 0.5 and σ = 0.5 per channel). No additional training, fine-tuning, or domain adaptation of any kind was performed on BRISC-2025.

Full classification metrics, including overall accuracy, macro-AUC, per-class precision, recall, and F1-score, along with normalized confusion matrices and FDT verification results, were reported on the external dataset. The rationale for this strict zero-shot protocol follows from the methodological literature on external validation in medical imaging: any form of adaptation on the external data would conflate model generalization with domain-specific optimization, undermining the validity of the external evaluation as an independent robustness test. The observed performance difference between internal and external evaluation provides a direct estimate of how much the learned representation degrades when exposed to different scanners, protocols, and institutional practices, information that is critical for any realistic assessment of clinical deployability.

## 4. Results

The experimental evaluation used two independent datasets. The internal dataset comprises 8040 post-contrast axial T1-weighted 2D brain MRI slices collected at Bandırma Onyedi Eylül University Hospital (glioma *n* = 1608; meningioma *n* = 1768; pituitary tumor *n* = 2251; no tumor *n* = 2413). The external dataset is BRISC-2025 (Brain Tumor MRI Dataset for Segmentation and Classification), a publicly available corpus of 6000 contrast-enhanced MRI slices (glioma *n* = 1401; meningioma *n* = 1635; pituitary tumor *n* = 1757; no tumor *n* = 1207) curated independently across acquisition sites, scanner vendors, and institutional protocols. Unlike the internal dataset, BRISC-2025 includes axial, coronal, and sagittal orientations annotated by expert neuroradiologists and released with an open-access DOI. The internal dataset supported both the stratified hold-out evaluation and five-fold stratified cross-validation, while BRISC-2025 was reserved exclusively for zero-shot external testing of the best-performing model (Swin-Tiny), applied without any fine-tuning, domain adaptation, or other model adjustment.

### 4.1. Overall Classification Performance

On the internal hold-out test split, the three architectures produced substantially different performance levels despite receiving identical preprocessing, augmentation, class-weighted loss, and optimization hyperparameters. The Pure CNN baseline achieved 86.24% overall accuracy, a macro-F1 of 85.67%, and a macro-AUC of 0.94. The Res-CNN improved on this, reaching 89.51% accuracy with a macro-F1 of 88.92% and a macro-AUC of 0.95. Both CNN baselines produced most of their errors in glioma versus pituitary confusion, consistent with these classes sharing overlapping enhancement characteristics on single axial slices [59].

The Swin-Tiny Transformer produced a clearly superior outcome. It achieved 97.42% test accuracy, a macro-F1 of 97.42%, and a macro-AUC of 0.99 on the same internal test partition. The absolute accuracy gap between Swin-Tiny and the best CNN baseline is approximately 8 percentage points (97.42% vs. 89.51%), a meaningful margin under identical experimental conditions. The macro-AUC of 0.99 indicates strong discriminative ability across operating thresholds, though it should be noted that AUC can remain high even with small calibration errors in near-ceiling regimes, and this metric alone does not guarantee well-calibrated posterior probabilities. Table 4 presents the overall performance summary across all three architectures.

Table 4 reports both macro-F1 and weighted-F1 to capture performance under class imbalance. Macro-F1 computes the F1-score independently for each class and then averages across classes with equal weight, so minority and majority classes contribute equally to the final value. This makes macro-F1 a more informative summary measure when class frequencies differ, as discussed in Section 3.8. In contrast, weighted-F1 averages per-class F1-scores using the number of test samples in each class as weights, which increases the influence of majority classes on the overall score.

To assess statistical significance, paired comparisons between Swin-Tiny and Res-CNN were conducted across cross-validation folds. The improvement in macro-F1 and accuracy was statistically significant (*p* < 0.05, paired *t*-test), indicating that the observed performance gain is unlikely to be due to random variation.

### 4.2. Training Dynamics and Convergence Behavior

Epoch-level training and validation metrics reveal distinct learning trajectories across the three architectures. Table 5, Table 6 and Table 7 report the detailed logs, while the summary analysis below highlights the main convergence patterns. The Pure CNN showed steady convergence across epochs, with training accuracy increasing from 40.58% at epoch 1 to 78.07% at epoch 10 and validation accuracy rising from 41.12% to 85.88%. Validation loss decreased monotonically from 1.2856 to 0.4627, indicating stable generalization rather than oscillatory behavior or early overfitting. Learning rate reductions at epochs 5 and 10 coincided with continued validation improvements, suggesting that the schedule supported refinement in later training. Despite this stability, the Pure CNN ultimately converged to a lower performance regime than the deeper Res-CNN and Swin-Tiny on the final test evaluation, consistent with limited representational capacity for this four-class slice-level task.

In Table 5 as reported the Pure CNN learned steadily across epochs, improving from 40.58% training accuracy and 41.12% validation accuracy at epoch 1 to 78.07% training accuracy and 85.88% validation accuracy by epoch 10. Validation loss dropped consistently from 1.2856 to 0.4627, which suggests stable generalization rather than overfitting within the shown training window. The learning rate reductions at epochs 5 and 10 coincided with continued improvements in validation performance, indicating that the schedule helped refine the model as training progressed. Table 5 reports the Pure CNN epoch log.

In Table 6 as seen, the Res-CNN learned rapidly in the early epochs, rising from 52.34% training accuracy and 55.78% validation accuracy at epoch 1 to 85.69% training accuracy and a peak validation accuracy of 88.55% at epoch 5. After epoch 5, validation loss increased from 0.4389 to 0.5216 and validation accuracy dropped to 86.12% by epoch 10, while training accuracy continued to improve to 91.45% and training loss kept decreasing. This divergence indicates the onset of overfitting. The Res-CNN’s deeper architecture (11.7 million parameters and 512-dimensional features) improves representation capacity compared to the Pure CNN, but without stronger global context modeling the extra depth yields limited validation gains in this slice-level classification setting. Table 6 reports the Res-CNN epoch log.

Table 7 displays Swin-Tiny converged quickly and maintained strong validation performance throughout training. Training accuracy increased from 89.15% at epoch 1 to 99.34% at epoch 7, while validation accuracy remained high, reaching 97.89% at epoch 3 and ending at 97.42% at epoch 7. Validation loss stayed low and stable (0.1542 at epoch 1, 0.0824 at epoch 3, and 0.0876 at epoch 7), suggesting good generalization with only minor fluctuations as the learning rate stepped down at epochs 5 and 7.

Using class-weighted cross-entropy improved minority-class recognition across all models, particularly for glioma and meningioma. Although the absolute class-weight values differed between the CNN baselines and Swin-Tiny due to normalization choices, their relative ordering remained consistent, with glioma receiving the highest weight and pituitary tumor the lowest, reflecting their frequencies in the training set. The benefit of weighting was most evident in Swin-Tiny, where per-class F1-scores remained tightly clustered around 97%, whereas the Res-CNN showed greater variation across classes. An auxiliary experiment without inverse-frequency weighting resulted in an approximately 3% to 4% reduction in macro-F1 across models, with the largest degradation observed for the glioma class. These results indicate that the weighting strategy improves minority-class recognition without changing the relative ranking of the evaluated architectures.

### 4.3. Per-Class Classification Performance

Per class results show that overall accuracy can hide meaningful differences in how each architecture performs on specific tumor types. Table 8 summarizes precision, recall, and F1 score for each class for all three models on the internal hold out test set.

The Pure CNN showed its weakest performance on glioma and meningioma, with F1-scores of 83.98% and 84.44%, respectively. These classes are visually similar on individual post-contrast T1 slices, and the Pure CNN appears to struggle with separating them based on local enhancement patterns alone. Performance improved for pituitary tumors (F1 = 87.04%) and was highest for the no tumor class (F1 = 87.22%), which benefits from the absence of enhancing lesions and therefore presents a clearer visual distinction from pathological cases. The overall pattern suggests that, while the Pure CNN generalizes stably, its limited receptive field and shallow feature hierarchy restrict its ability to capture broader anatomical context needed for fine-grained tumor differentiation.

The Res-CNN improved per class performance across all tumor types relative to the Pure CNN. Glioma F1-score increased to 87.61%, and meningioma and pituitary tumor F1-scores reached 88.44% and 89.57%, respectively. The no tumor class achieved the highest Res-CNN F1-score at 90.05%. Unlike the earlier unstable behavior observed during training, the test set results show a more balanced precision recall tradeoff across classes, indicating that the deeper architecture enhances representational capacity without introducing severe class-specific bias at inference time.

Swin-Tiny achieved the most consistent per-class performance, with F1-scores tightly clustered between 96.98% and 97.74% across all four classes. Glioma and meningioma performance remained nearly symmetric, and no tumor detection showed no degradation despite class imbalance. This uniformity across classes with different training frequencies supports the effectiveness of combining inverse frequency class weighting with the Swin architecture’s global attention mechanism. Minor residual confusion between glioma and meningioma persists, as reflected by slightly higher meningioma recall than precision, which aligns with the known radiologic overlap between these tumor types.

### 4.4. Confusion Matrix Analysis and Error Patterns

Normalized confusion matrices on the internal test set showed clearly different error profiles across the three architectures. The Pure CNN made more cross-class confusions than the deeper models, with its errors clustering most strongly between glioma and meningioma, which share overlapping enhancement patterns on post-contrast T1 slices. A second recurring mistake involved confusing pituitary tumor slices with the no tumor class, consistent with missed or subtle sellar region enhancement when lesions are small, centrally located, or partially captured in a single slice. These patterns suggest that the Pure CNN relies heavily on local texture cues and struggles to integrate broader anatomical context, which increases confusion for visually similar tumor types and for subtle midline findings.

The Res-CNN reduced some inter-class confusion relative to the Pure CNN but introduced a different failure mode: a strong bias toward the meningioma class at the expense of glioma detection. Although the Res-CNN correctly classified more meningioma slices (recall = 82.44%), it achieved this partly by over-predicting meningioma for cases that were actually gliomas. The net effect was a trade-off that improved aggregate accuracy by 3.27 percentage points over the Pure CNN (89.51% vs. 86.24%) without fully resolving the core difficulty of glioma–pituitary discrimination.

The Swin-Tiny confusion matrix was dominated by diagonal entries, with off-diagonal values near zero. On the internal test set, a very small number of glioma slices were incorrectly labeled as meningioma, and the remaining off-diagonal entries were negligible. This residual glioma–meningioma confusion, while minimal in absolute count, is consistent with known neuroradiologic overlap: dural-based meningiomas with abundant peritumoral edema and heterogeneous enhancement can closely resemble dural-contacting gliomas on a single axial T1-CE slice, particularly when key discriminators such as the dural tail sign, a CSF cleft, or patient demographic information are unavailable. The persistence of this specific confusion pattern even in a near-perfect classifier suggests that it reflects genuine ambiguity in the imaging data rather than a learnable pattern that the model failed to capture.

### 4.5. Cross-Validation Results

Five-fold stratified cross-validation on the full internal dataset confirmed the stability of Swin-Tiny and provided variance estimates that are not available from a single hold-out split. Table 9 reports the fold-level and summary statistics. Based on fold-to-fold variability, the approximate 95% confidence interval for accuracy was 96.9% to 97.9%, further supporting the stability of the model across different partitions.

As seen from Table 9 the mean cross-validation accuracy was 97.40% ± 0.28%, with a macro-AUC of 0.9958 ± 0.0004. The fold-to-fold standard deviation indicates that performance remains stable across different stratified partitions, which reduces the likelihood that the hold-out result reflects an unusually favorable split. The cross-validation mean is close to the internal hold-out performance, which is expected because each fold evaluates the model on a different subset while training on 80% of the data.

Per-class F1-scores also showed low variance across folds. Glioma F1 ranged from 96.94% to 97.60% (mean 97.30% ± 0.28%), while meningioma ranged from 96.88% to 97.46% (mean 97.24% ± 0.23%). Pituitary tumor performance was similarly consistent, ranging from 97.42% to 97.93% (mean 97.72% ± 0.20%). This consistency supports the claim that Swin-Tiny learns class-discriminative representations that generalize across partitions rather than depending on a particular split. The caveat about potential intra-patient leakage in slice-level splitting remains important: adjacent slices from the same scan can appear in both training and evaluation folds, and this risk cannot be fully removed without patient identifiers. External evaluation, where leakage cannot occur, provides the stronger test of generalization.

### 4.6. External Validation on BRISC-2025

Zero-shot inference on the BRISC-2025 dataset used the best Swin-Tiny checkpoint selected by internal validation macro-F1 and was applied directly, with no fine tuning or domain adaptation. The model achieved 94.82% accuracy with a macro-AUC of 0.97. Table 10 compares internal and external performance. Bootstrapped 95% confidence intervals for the external accuracy were computed using 1000 resamples and are reported alongside the 94.82%-point estimate.

As shown in Table 10, accuracy decreased by 2.58 percentage points when moving from the internal cross-validation mean to BRISC-2025, dropping from 97.40% ± 0.28 to 94.82%. Macro-AUC declined from 0.9958 ± 0.0004 internally to 0.97 externally, indicating a measurable reduction in ranking performance under distribution shift while remaining high overall. The magnitude of this degradation is modest relative to many external validation studies in medical imaging, where cross-institution accuracy drops of 5 to 15 percentage points are commonly reported. The comparatively smaller drop observed here is consistent with Swin-Tiny learning features that generalize across acquisition environments, although it may also reflect similarities in imaging modality and label space between the internal dataset and BRISC-2025.

Per-class results indicate that glioma (F1 = 94.10%) and meningioma (F1 = 93.60%) experienced the largest reductions relative to internal performance. These classes are the most visually ambiguous on single post-contrast T1 slices and are therefore more sensitive to variations in scanner characteristics, contrast timing, and slice orientation. In contrast, pituitary tumor (F1 = 95.50%) and no tumor (F1 = 96.10%) maintained higher external F1-scores, likely because their visual signatures, a centrally located sellar mass and the absence of enhancing lesions, are more robust to acquisition variability. BRISC-2025 includes slices from multiple anatomical orientations, all processed identically as 2D inputs during evaluation, which introduces additional variability not present in the internal training data and further explains the observed performance gap.

Despite this shift, glioma–meningioma confusion remained the dominant residual error mode on BRISC-2025, mirroring the internal evaluation. The external confusion matrix shows elevated off-diagonal entries primarily between these two classes, with comparatively fewer errors elsewhere. The persistence of this pattern across datasets acquired with different scanners and protocols suggests that the remaining errors reflect genuine radiologic overlap rather than dataset-specific artifacts.

### 4.7. Model Complexity and Computational Cost

Table 11 reports model size, computational cost (multiply–accumulate operations, MACs), training time, and inference latency for each architecture. All measurements were obtained on a single NVIDIA Tesla T4 GPU (16 GB VRAM, Turing architecture) to ensure comparability.

The key observation from Table 11 is that accuracy gains do not scale linearly with computational investment. Increasing model size from 1.3 M to 11.7 M parameters (Pure CNN to Res-CNN) improved test accuracy from 86.24% to 89.51%, a gain of 3.27 percentage points, which corresponds to roughly 0.31 points per million additional parameters. Increasing model size further from 11.7 M to 28.3 M parameters (Res-CNN to Swin-Tiny) improved accuracy from 89.51% to 97.42%, a gain of 7.91 percentage points, or about 0.48 points per million additional parameters. These differences are not explained by parameter count alone and instead reflect architectural inductive bias. The convolutional baselines emphasize local receptive fields, while Swin-Tiny uses shifted-window self-attention to incorporate broader spatial context, which is better aligned with slice-level tumor discrimination. Swin-Tiny’s inference latency of about 35 ms per slice remains practical for retrospective batch processing and offline clinical decision support, but it may be restrictive for real-time interactive workflows.

### 4.8. Formal Decision Trace Verification Results

The FDT and SMT verification pipeline was applied to all Swin-Tiny predictions on both the internal test set and BRISC 2025. Table 12 summarizes the verification outcomes. All timing was measured on the Tesla T4 GPU as the average wall-clock time over 100 independent trials per dataset.

On the internal test set, 98.76% of Swin-Tiny predictions received an SAT result from the Z3 solver, meaning the predicted class was consistent with all encoded neuroradiologic constraints given the extracted symbolic features. The remaining 1.24% received UNSAT results. To distinguish between benign and diagnostically informative violations, we cross-referenced each UNSAT case with the ground-truth label. Of the 1.24% UNSAT cases, 1.11% involved predictions that were correct according to the ground truth but failed a constraint due to borderline symbolic feature values, while only 0.13% involved both a constraint violation and an incorrect classification. This breakdown confirms that the majority of UNSAT results reflect sensitivity of the rule-based verification to discrete threshold boundaries rather than genuine diagnostic errors by the model.

A concrete example illustrates this phenomenon. One correctly predicted glioma slice received UNSAT because the pixel intensity variance within the Otsu-segmented region was 0.098, falling just below the 0.1 threshold encoding the “heterogeneous enhancement present” constraint. The model identified the slice as glioma based on learned visual features that incorporate context beyond raw intensity variance, but the symbolic verification system, operating on a single hand-crafted threshold, flagged the prediction as constraint-violating. This case highlights both the value and the inherent limitation of rule-based post hoc verification: it catches genuine constraint failures but can also flag near-boundary cases where the continuous feature distribution does not align cleanly with discrete symbolic thresholds.

On BRISC 2025, the constraint violation rate increased to 2.88%. Of these, 2.34% were correct predictions with borderline features, and 0.54% were both UNSAT and misclassified. The increase relative to the internal set is consistent with the expectation that slices acquired under different scanner vendors and contrast protocols produce symbolic features whose distributions shift relative to the internally calibrated thresholds. The higher UNSAT rate on external data does not reflect a proportional increase in classification errors but rather a widening gap between the fixed constraint thresholds and the shifted feature distributions of the external data.

Dual-channel agreement between the Z3 solver and the independent Python Boolean evaluator was 100% on both datasets, confirming that the SMT encoding faithfully represents the intended logical constraints with no encoding bugs detected. Combined with inference latency (~35 ms), symbolic feature extraction (~10 ms), and solver time (~12.5 ms), the total pipeline latency was approximately 47–48 ms per slice on the Tesla T4 GPU, within acceptable bounds for offline clinical decision support.

### 4.9. Clinical Relevance of Residual Misclassifications

From a clinical standpoint, the most consequential residual errors involved confusion between meningiomas and gliomas, a diagnostic dilemma that is well recognized in neuroradiology. Dural-based meningiomas with abundant peritumoral edema and heterogeneous enhancement can closely resemble dural-abutting gliomas when only a single axial T1-weighted post-contrast slice is available. This resemblance is a genuine property of the imaging data, not an artifact of the classification framework, and it is unlikely to be resolved without access to additional imaging sequences (T2, FLAIR), volumetric context, or clinical metadata.

The clinical stakes of this confusion pattern are asymmetric. Meningiomas are often managed with observation or local surgical resection, while high-grade gliomas require prompt histologic confirmation and initiation of multimodal chemoradiation. A false-negative glioma classification (glioma misclassified as meningioma) could delay necessary treatment, while a false-positive glioma classification (meningioma misclassified as glioma) could trigger unnecessary invasive procedures. Swin-Tiny’s ability to reduce this confusion to a small number of cases on the internal test set, and to maintain comparably low rates on the BRISC-2025 external set, represents a meaningful improvement over the CNN baselines where glioma–meningioma misclassification was pervasive. Nevertheless, the residual error rate confirms that slice-level classification alone is insufficient for confident clinical diagnosis and must be integrated into a broader diagnostic workflow that includes radiologic review, multi-sequence imaging, and histopathologic confirmation where indicated.

## 5. Discussion

This study compared a simple custom CNN baseline, a Res-CNN variant, and a Swin-Tiny Transformer for the classification of brain tumors from T1-weighted contrast-enhanced MRI slices. The Swin-Tiny model achieved substantially higher accuracy, reaching 97.42% on the internal test set, 97.40% ± 0.28% on five-fold cross-validation, and 94.82% on the external BRISC-2025 dataset, compared with the Pure CNN and Res-CNN baselines, which achieved accuracies in the range of approximately 86% to 89%. This improvement was accompanied by a clear advantage in macro-AUC, with Swin-Tiny achieving 0.994 versus 0.94 to 0.95 for the CNN baselines, as well as more consistent per-class F1-scores. The persistence of this performance gap across both internal and external evaluations suggests that Swin-Tiny captures tumor-relevant patterns more effectively than convolutional architectures within the constraints of 2D axial slice classification.

The hierarchical shifted-window attention mechanism of the Swin Transformer appears to be a key driver of this performance advantage. Unlike conventional CNNs, whose receptive fields expand only gradually with network depth, transformer-based architectures can directly model relationships between spatially distant but functionally related regions within a single forward pass. This capability enables integration of local tumor appearance, such as heterogeneous enhancement, with broader contextual cues, including peritumoral edema and mass effect on adjacent structures. Transfer learning from ImageNet pretraining further contributed to faster convergence and more effective feature learning for Swin-Tiny, an advantage not available to the Pure CNN, which was trained entirely from scratch on a comparatively limited internal dataset.

A methodological strength of this work is the combination of five-fold stratified cross-validation on the internal clinical dataset with external validation on an independently curated dataset, BRISC-2025, which includes images acquired across different scanners, sites, and protocols. The consistency of Swin-Tiny performance across cross-validation folds, with a mean accuracy of (mean 97.40% ± 0.28%) and its controlled decrease to 94.82% on BRISC-2025 support robustness to distributional shift and suggest genuine learning of generalizable tumor-class associations rather than memorization of training samples. This observation aligns with recent reviews reporting improved generalization of transformer-based models in medical imaging when evaluated under rigorous validation protocols.

All three models benefited from inverse-frequency class-weighted loss, which increased the gradient contribution of minority classes during training. However, Swin-Tiny exhibited markedly more balanced per-class performance, with F1-scores tightly clustered around 97% to 98% on the internal test set, whereas the Res-CNN showed greater variability across classes. This indicates that architectural properties, rather than class weighting alone, contribute to improved robustness under class imbalance. Global attention mechanisms may allow sparse diagnostic cues for minority classes to be aggregated across the image, whereas convolutional models rely more heavily on locally clustered patterns.

Despite near-ceiling overall performance, confusion matrix analysis revealed that confusion between glioma and meningioma remained the dominant residual error mode on both the internal test set and BRISC-2025. This behavior is consistent with known neuroradiologic overlap, as dural-based meningiomas with surrounding edema and heterogeneous enhancement can closely resemble infiltrative gliomas on a single axial post-contrast T1 slice, particularly in the absence of discriminative features such as a dural tail, cerebrospinal fluid cleft, or patient demographic information. These residual errors reflect intrinsic ambiguity in slice-level classification rather than a failure of the model and may require multi-slice context, additional imaging sequences, or clinical metadata for confident resolution.

Grad-CAM visualizations confirmed that the Swin Transformer consistently localized activation to tumor-bearing regions and highlighted anatomically plausible intensity and structural features across tumor types. While these visualizations support the interpretability of the learned representations, they remain qualitative and do not quantify diagnostic confidence or inter-observer agreement. Formal reader studies would therefore be required to determine whether Swin-Tiny predictions improve radiologist confidence or reduce variability in clinical practice.

From a computational perspective, Swin-Tiny requires approximately 28.3 million parameters and around two hours of training on a single Tesla T4 GPU, compared with approximately 1.3 million parameters for the Pure CNN and 11.7 million for the Res-CNN, both of which require between one and one and a half hours of training. Inference latency for Swin-Tiny is approximately 30 to 50 milliseconds per slice on a Tesla T4 GPU, which is acceptable for retrospective analysis and screening workflows but may be limiting for real-time interactive reporting. This computational overhead presents a practical barrier in resource-constrained clinical environments where high-end GPUs are not readily available.

Potential mitigation strategies include knowledge distillation, in which a smaller student model is trained to approximate Swin-Tiny outputs, model quantization to reduce numerical precision, or hybrid architectures that restrict attention mechanisms to diagnostically salient regions. Such approaches may improve deployability but typically involve accuracy trade-offs that require careful validation prior to clinical adoption.

An important caveat is that both the internal clinical dataset and BRISC-2025 consist of individual 2D slices without patient identifiers, which prevents patient-wise stratification and creates the possibility that correlated slices may appear across different folds during cross-validation. In routine clinical practice, neuroradiologists interpret full 3D volumes with multiplanar reconstructions and integrate imaging findings with clinical history, laboratory results, and histopathology. Accordingly, slice-level accuracy approaching 98% should not be interpreted as patient-level diagnostic performance, but rather as strong performance on a constrained and well-defined benchmark task. Several published studies on similar public slice-level datasets have reported comparably high accuracies using both CNN and transformer architectures, suggesting that performance on such benchmarks may be approaching saturation. Demonstrating stronger clinical relevance will likely require patient-wise validation, prospective evaluation on consecutive clinical cases, and direct comparison with expert neuroradiologist performance.

The observed performance gap between Swin-Tiny and the CNN baselines must also be interpreted with caution. Although architectural differences likely contribute, Swin-Tiny benefits from ImageNet pretraining, whereas the CNN baselines were trained from scratch. This difference may account for part of the observed improvement. Future work should include pretrained CNN baselines and transformer models trained from scratch to better disentangle architectural effects from pretraining advantages.

### 5.1. Limitations

This study has several limitations. First, both the internal clinical dataset and the BRISC-2025 dataset lack patient identifiers, which makes patient-wise validation impossible and introduces the possibility that multiple slices from the same patient may appear across different folds during cross-validation. This limitation is common in slice-level MRI datasets and does not invalidate the reported results, but it does indicate the need for external patient-level validation using prospectively collected data. In routine clinical practice, neuroradiologists evaluate the full three-dimensional MRI volume rather than individual slices, and slice-level performance approaching 98 to 99 percent accuracy does not necessarily translate directly to patient-level diagnostic accuracy.

Second, the study was restricted to axial post-contrast T1-weighted MRI slices, excluding other routinely used sequences such as T2-weighted and FLAIR imaging, as well as three-dimensional volumetric information. Radiologists integrate information across multiple sequences and planes when forming diagnoses, and limiting the input to a single modality and orientation may simplify the classification task. As a result, conclusions about performance in fully multimodal clinical scenarios remain limited.

Third, because fold-level training logs and code for the CNN baselines were not retained, formal paired statistical significance testing, such as McNemar’s test or paired *t* tests, could not be performed to quantify performance differences between the CNN and Swin-Tiny models. Although the observed accuracy gap of approximately eight percentage points is substantial and consistent across evaluations, formal hypothesis testing would have provided additional statistical support.

Fourth, deployment of Swin-Tiny in clinical environments is constrained by its computational requirements. While the model achieves strong performance, its reliance on GPU acceleration limits immediate applicability in resource-constrained settings, including hospitals without access to dedicated high-performance hardware.

Finally, although BRISC-2025 provides an independent external test set, it contains the same four diagnostic classes and similar axial post-contrast T1 slices as the internal dataset. Validation on more diverse cohorts, including data from different geographic regions, scanner vendors, and acquisition protocols, would provide stronger evidence of generalization across real-world clinical settings.

### 5.2. Future Directions

Future work should prioritize patient-centered validation through patient-wise stratification and prospective evaluation of individual cases to better assess clinical usefulness and agreement with neuroradiologist interpretations. Incorporating multimodal and volumetric modeling may further improve performance and generalizability by integrating multiple MRI sequences and leveraging three-dimensional spatial context through 3D convolutional or recurrent architectures. Transformer-based models are particularly well suited to such multimodal inputs and may benefit from cross-attention mechanisms to fuse information across sequences and planes.

In addition, model compression, lightweight architectures, neural architecture search, and hybrid CNN and Transformer designs will be important for enabling deployment on edge devices and in resource-constrained clinical environments. Continued investigation of explainability methods, together with close collaboration with neuroradiologists, will help clarify the clinical utility and decision-making value of these systems. Finally, extending the current evaluation framework, including cross-validation, external validation, and Grad-CAM analysis, to other neuroradiologic tasks and to imaging of additional organs may support broader generalization across clinical applications and promote wider adoption of trustworthy AI tools in medical imaging.

## 6. Conclusions

This study presents a deep learning framework augmented with formal verification for four-class brain tumor classification from contrast-enhanced axial T1-weighted MRI slices, integrating a fine-tuned Swin-Tiny Transformer with inverse-frequency class-weighted learning and an SMT-based formal decision-trace verification layer. Under tightly controlled experimental conditions, including identical preprocessing, augmentation, optimization, and evaluation protocols, the Swin-Tiny architecture substantially outperformed carefully matched convolutional baselines, achieving 97.42% slice-level accuracy (macro-F1 97.42%, macro-AUC 0.994) on the Bandırma Onyedi Eylül University Hospital dataset (*n* = 8040 slices) and maintaining strong generalization at 94.82% accuracy (macro-AUC 0.97) in zero-shot evaluation on the independent BRISC-2025 external corpus. Five-fold stratified cross-validation further confirmed the internal robustness of the Swin-Tiny model (mean accuracy 97.40% ± 0.28%).

The observed performance advantage, approximately eight percentage points over the strongest residual CNN baseline (97.42% vs. 89.51%), underscores the value of hierarchical shifted-window self-attention for slice-level neuro-oncologic image recognition. Unlike purely convolutional architectures, which accumulate long-range context only indirectly through depth and pooling, the Swin Transformer explicitly models spatially distributed diagnostic cues, including enhancement heterogeneity, anatomical proximity, and mass-effect signatures, within a single forward pass. This inductive bias appears particularly well suited to the constrained 2D setting, where volumetric continuity, multi-sequence information, and patient metadata are unavailable. The persistent yet greatly reduced glioma–meningioma confusion even at near-ceiling accuracy reflects genuine radiologic overlap rather than model deficiency, highlighting an intrinsic limitation of single-slice post-contrast T1 classification.

The incorporation of SMT-verified formal decision traces constitutes a meaningful step toward clinical accountability. By encoding domain-grounded constraints, such as heterogeneous enhancement, sellar proximity, and dural-contact proxies, and producing machine-checkable certificates, the framework enables post hoc logical auditing of predictions, a desirable property for high-stakes diagnostic support systems. Although constraint-violation rates remained low (1.24–2.88%) and were dominated by borderline feature cases rather than outright misclassifications, the verification layer provides a transparent, auditable complement to probabilistic outputs and gradient-based attribution maps.

Several limitations temper the interpretation of these results. First, the absence of patient identifiers in both datasets precludes patient-wise stratification, raising the possibility of intra-patient slice correlation and optimistic internal performance estimates. Second, restriction to axial post-contrast T1 slices excludes complementary sequences (T2, FLAIR, perfusion, diffusion) and 3D context routinely used by neuroradiologists. Third, while external validation on BRISC-2025 offers evidence of robustness to acquisition-domain shift, broader multi-institutional, multi-vendor, and multi-orientation testing remains necessary before claims of general clinical utility can be made. Finally, the computational footprint of Swin-Tiny (approximately 28 million parameters and approximately 35 ms per slice inference on a Tesla T4 GPU) constrains immediate deployment in resource-limited settings, motivating future work on distillation, quantization, or hybrid lightweight designs.

Taken together, the findings demonstrate that hierarchical vision transformers, when rigorously benchmarked and augmented with formal verification, can deliver highly accurate and traceable slice-level brain tumor recognition under severe informational constraints. These results strengthen the case for attention-based inductive biases in medical imaging tasks that require integration of local texture with global anatomical context. Nonetheless, translation to patient-level diagnosis will require prospective, multimodal, patient-stratified validation, integration with volumetric and longitudinal data, and head-to-head comparison against expert neuroradiologist performance in real-world clinical workflows.

Future efforts should therefore prioritize patient-centric evaluation protocols, multimodal and volumetric architectures that better approximate clinical reasoning, efficient transformer variants or state-space models for edge deployment, and tighter clinician–AI collaboration to define clinically actionable verification constraints and decision-support interfaces. By addressing these directions, transformer-based frameworks augmented with formal verification can move closer to supporting, not supplanting, expert neuroradiologic interpretation in the management of primary brain tumors.

This work contributes an empirically strong, methodologically careful benchmark and a prototype accountability mechanism that may help guide the next generation of trustworthy AI tools in neuro-oncology imaging.

## Figures and Tables

**Table 1:** Summary of dataset characteristics used in this study.

Characteristic	Glioma	Meningioma	Pituitary	No Tumor
Internal (*n* = 8040)	1608 (20.0%)	1768 (22.0%)	2251 (28.0%)	2413 (30.0%)
External (*n* = 6000)	1401	1635	1757	1207
Imaging protocol	Post-contrast axial T1-weighted, gadolinium-enhanced (TE 10–15 ms, TR 500–800 ms, 1.5 T/3 T)			
Patient identifiers	Not available in either dataset			
Unit of analysis	Individual 2D axial slice			

**Table 2:** Architectural specifications of the three evaluated models.

Property	Pure CNN	Res-CNN	Swin-Tiny
Parameters	~1.3 M	~11.7 M	28.3 M
Input size	224 × 224 × 3	224 × 224 × 3	224 × 224 × 3
Pretrained	No	No	ImageNet-1K
Blocks/Stages	2 conv blocks	4 residual stages	4 Swin stages
Feature dim.	64	512	768
Dropout	0.5	0.5	N/A
Classifier	FC(512) + Linear(4)	FC(512) + Linear(4)	Linear(4)
Context modeling	Local (small RF)	Local (larger RF)	Local + global (shifted windows)

**Table 3:** Training hyperparameters applied uniformly across all three models.

Hyperparameter	Value
Optimizer	AdamW (β_1_ = 0.9, β_2_ = 0.999)
Initial learning rate	3 × 10^−4^
Weight decay	1 × 10^−4^
Batch size	32
Max epochs	20
Early stopping metric	Validation macro-F1
Early stopping patience	5 epochs
LR scheduler	ReduceLROnPlateau (factor = 0.5, patience = 2)
Mixed precision	FP16 (torch.amp)
Loss function	Weighted cross-entropy (inverse freq.)
Hardware	Single NVIDIA Tesla T4 GPU

**Table 4:** Overall classification performance on the internal hold-out test split. Bold values indicate the best result per metric.

Metric	Pure CNN	Res-CNN	Swin-Tiny
Accuracy (%)	86.24	89.51	97.42
Macro-F1 (%)	85.67	88.92	97.42
Weighted-F1 (%)	86.05	89.34	97.63
Macro-AUC	0.94	0.95	0.994

**Table 5:** Pure CNN training log with all experiments conducted on a single NVIDIA Tesla T4 GPU.

Epoch	Train Loss	Train Acc (%)	Val. Loss	Val. Acc (%)	Learning Rate
1	1.3412	40.58	1.2856	41.12	3.0 × 10^−4^
3	1.1547	55.23	0.9821	60.45	3.0 × 10^−4^
5	1.0382	64.18	0.7453	73.31	1.5 × 10^−4^
10	0.8914	78.07	0.4627	85.88	7.5 × 10^−5^

**Table 6:** Res-CNN training log (selected epochs). Single NVIDIA Tesla T4 GPU.

Epoch	Train Loss	Train Acc (%)	Val. Loss	Val. Acc (%)	Learning Rate
1	1.1823	52.34	1.0945	55.78	3.0 × 10^−4^
3	0.8741	72.56	0.6924	74.89	3.0 × 10^−4^
5	0.6235	85.69	0.4389	88.55	1.5 × 10^−4^
10	0.3912	91.45	0.5216	86.12	3.75 × 10^−5^

**Table 7:** Swin-Tiny Transformer training log (selected epochs). Single NVIDIA Tesla T4 GPU.

Epoch	Train Loss	Train Acc (%)	Val. Loss	Val. Acc (%)	Learning Rate
1	0.3421	89.15	0.1542	95.67	3.0 × 10^−4^
3	0.0812	97.43	0.0824	97.89	3.0 × 10^−4^
5	0.0385	98.97	0.0931	97.51	1.5 × 10^−4^
7	0.0218	99.34	0.0876	97.42	7.5 × 10^−5^

**Table 8:** Per-class precision, recall, and F1-score (%) on the internal hold-out test set.

Model	Class	Precision	Recall	F1-Score	Support
Pure CNN	Glioma	85.20	82.80	83.98	321
	Meningioma	85.40	83.50	84.44	353
	Pituitary	88.00	86.10	87.04	450
	No Tumor	88.60	86.80	87.22	482
Res-CNN	Glioma	88.31	86.92	87.61	321
	Meningioma	89.04	87.85	88.44	353
	Pituitary	90.11	89.03	89.57	450
	No Tumor	91.02	89.91	90.05	482
Swin-Tiny	Glioma	97.12	96.84	96.98	321
	Meningioma	97.43	97.19	97.40	353
	Pituitary	97.91	97.58	97.74	450
	No Tumor	97.81	97.63	97.65	482

**Table 9:** Five-fold stratified cross-validation results for Swin-Tiny on the internal dataset (8040 slices). F1-No Tumor column omitted for space; all folds exceeded 97.5%.

Fold	Accuracy (%)	Macro-AUC	F1-Glioma	F1-Mening.	F1-Pituitary
1	97.18	0.9956	97.10	97.28	97.65
2	97.62	0.9961	97.55	97.40	97.88
3	97.44	0.9958	97.32	97.19	97.74
4	97.05	0.9952	96.94	96.88	97.42
5	97.71	0.9963	97.60	97.46	97.93
Mean ± SD	97.40 ± 0.28	0.9958 ± 0.0004	97.30 ± 0.28	97.24 ± 0.23	97.72 ± 0.20

**Table 10:** Swin-Tiny performance: internal evaluation vs. external validation on BRISC-2025.

Metric	Internal (CV Mean ± SD)	External (BRISC-2025)
Accuracy (%)	97.40 ± 0.28	94.82 [94.37, 95.27]
Macro-AUC	0.9958 ± 0.0004	0.97
F1-Glioma (%)	97.30 ± 0.28	94.10
F1-Meningioma (%)	97.24 ± 0.23	93.60
F1-Pituitary (%)	97.72 ± 0.20	95.50
F1-No Tumor (%)	97.70 ± 0.18	96.10

**Table 11:** Model complexity and computational cost.

Property	Pure CNN	Res-CNN	Swin-Tiny
Parameters (M)	1.3	11.7	28.3
MACs (G)	0.42	1.82	4.51
Training time (h)	~1.0	~1.5	~2.0
Inference (ms/slice)	~5	~12	~35
Test Accuracy (%)	86.24	89.51	97.42

**Table 12:** SMT verification results for Swin-Tiny predictions. All latency measured on a single NVIDIA Tesla T4 GPU.

Verification Metric	Internal Test Set	BRISC 2025
Traces verified (SAT)	98.76%	97.12%
Violations (UNSAT)	1.24%	2.88%
UNSAT with correct label	1.11%	2.34%
UNSAT with incorrect label	0.13%	0.54%
Dual-channel agreement	100.0%	100.0%
Mean verification latency	12.4 ms/slice	12.7 ms/slice
Total pipeline latency	~47 ms/slice	~48 ms/slice

## Data Availability

Data Availability Statement: The internal dataset used in this study is not publicly available due to institutional restrictions. The external dataset (BRISC-2025) is publicly available and was used for independent validation.
